# Occupational Stress, Nutritional Health, and Physical Pain Among Hong Kong Schoolteachers: A Cross-Sectional Study

**DOI:** 10.3390/nu18182956

**Published:** 2026-09-09

**Authors:** Kenneth K. H. Lo, Yiting Weng, Jiali He, Raymond C. K. Chung, Wendy W. Y. So, Jessie Ho, Man-sau Wong, Hector W. H. Tsang

**Affiliations:** 1 Department of Food Science and Nutrition, The Hong Kong Polytechnic University, Hung Hom, Kowloon, Hong Kong, China; kenneth.kh.lo@polyu.edu.hk (K.K.H.L.);; 2 Research Centre for Chinese Medicine Innovation, The Hong Kong Polytechnic University, Hung Hom, Kowloon, Hong Kong, China; 3 Department of Rehabilitation Sciences, The Hong Kong Polytechnic University, Hung Hom, Kowloon, Hong Kong, China; 4 The Hong Kong Jockey Club Centre for Suicide Research and Prevention, The University of Hong Kong, Sai Ying Pun, Hong Kong Island, Hong Kong, China; 5 Mental Health Research Centre, The Hong Kong Polytechnic University, Hung Hom, Kowloon, Hong Kong, China

**Keywords:** occupational stress, nutritional health, physical pain, schoolteachers

## Abstract

**Background/Objectives**: Teaching is a highly stressful occupation, yet the associations between occupational stress, nutritional health, and physical pain among schoolteachers remain understudied, particularly in non-Western contexts. This study examined whether high occupational stress is associated with reduced favorable dietary behaviors, as well as with the presence and prolonged duration of physical pain among Hong Kong schoolteachers. **Methods**: Cross-sectional data were analyzed from 267 secondary and special schoolteachers (65.5% female, mean age 41.5 years) who completed the Occupational Stress Indicator-2 (OSI-2), a dietary behavior questionnaire, and a region-specific pain assessment. One-way analysis of variance, independent t-tests, and linear regression (adjusting for age, gender, income, school type, depression, anxiety, stress, and sleep quality) were conducted. **Results**: In the adjusted linear regression, moderate red meat intake was associated with lower odds of high occupational stress. Shoulder, forearm/wrist, and hip pain were associated with high OSI-2 scores in univariable analyses. However, only pain duration of >30 days and depression remained significant in fully adjusted models. No other dietary variables or pain locations showed significant associations. **Conclusions**: Occupational stress among Hong Kong schoolteachers is independently associated with prolonged musculoskeletal pain, with depression strongly confounding the pain–stress relationship. An integrated approach is necessary to address the direct physical, nutritional, and mental health consequences of occupational stress.

## 1. Introduction

Teaching is widely recognized as one of the most highly stressful occupations worldwide [1]. A scoping review of 40 studies found that the prevalence of clinically meaningful stress among teachers ranges from 8.3% to 87.1%, burnout from 25.1% to 74%, anxiety from 38% to 41.2%, and depression from 4% to 77% [1]. Another meta-analysis of 54 studies synthesizing data from 256,896 teachers across 22 countries reported a pooled prevalence of stress at 62.6%, anxiety at 36.3%, and depression at 59.9% among teachers during the COVID-19 pandemic [2]. Following the COVID-19 outbreak, teachers devoted considerable time and effort not only to adapting personally but also to helping their students adjust to remote classes [3]. Beyond the traditional tasks of daily teaching, teachers are involved in various other duties, including curriculum assessment, extracurricular activity organization, and administrative tasks [4,5]. Some of these duties, such as supporting the behavioral and emotional needs of diverse students, significantly increase teacher stress [6]. These stressors have systemic consequences because high teacher stress and burnout are associated with reduced teaching quality, low academic achievement among students, and increased teacher attrition [7,8].

Another important but understudied consequence of chronic occupational stress is its potential impact on dietary behavior. For instance, individuals often resort to overeating after being exposed to forbidden foods during dietary modifications because their cravings for restricted items intensify [9], particularly for calorie-dense and proinflammatory comfort foods (e.g., confectionery and fried foods) as part of the body’s adaptation mechanism [10]. Overeating is an unhealthy coping mechanism that provides relief from negative emotions, such as distress, anxiety, and depression; subsequently, this behavior worsens obesity [11,12].

Beyond nutritional health, the teaching occupation is also closely linked to adverse physical pain, particularly musculoskeletal disorders (MSDs). A meta-analysis reported that the prevalence of MSDs among teachers ranges between 39% and 95%, with neck and low back pain being the most common regions [13]. A study of Chinese teachers found that self-reported neck and shoulder pain is associated with prolonged standing, prolonged sitting, and static posture [14]. Notably, work stress has been identified as a cause of significant musculoskeletal system pain in the body as a whole and in the lumbar, neck, and back regions in particular; moreover, schoolteachers’ increased job-related stress has a direct impact on the occurrence of musculoskeletal pain [15,16]. A cross-sectional study of secondary school teachers found that work-related psychosocial factors and psychological distress were significantly associated with self-reported low back pain and neck/shoulder pain, with psychological distress mediating the relationship between work factors and pain [17]. Depression and anxiety frequently co-occur with chronic pain, each exacerbating the other in a bidirectional manner [18].

While some studies have examined the relationship between occupational stress, diet, and physical pain, a comprehensive investigation among schoolteachers remains lacking. We addressed this gap by analyzing cross-sectional data from the Mental, Exercise, and Diet Health (MED) project, which was conducted in Hong Kong secondary and special schools during the COVID-19 pandemic. We analyzed this dataset to gain deeper insights into how occupational stress affects nutritional health and physical pain in a high-stress working population during a particularly challenging period.

## 2. Materials and Methods

### 2.1. Study Design and Participants

This cross-sectional study used baseline data from the MED project conducted in Hong Kong. Ethical approval was granted by the Human Subjects Ethics Subcommittee of The Hong Kong Polytechnic University (reference number: HSEARS20221213001). Teachers were recruited from 50 secondary schools and nine special schools between June 2022 and June 2024. All participants provided written informed consent before completing an online self-report questionnaire. For the present analysis, we included 267 teachers with complete data on occupational stress, dietary habits, physical pain, and covariates.

### 2.2. Measures

Occupational stress: The primary exposure was occupational stress, which was measured using the Chinese version of the Occupational Stress Indicator-2 (OSI-2). The OSI-2 consists of 40 items assessing perceived sources of job-related stress. Each item is rated on a six-point Likert scale (1 = very definitely not a source to 6 = very definitely a source). Total scores range from 40 to 240, with higher scores indicating greater occupational stress.

Nutritional health: Dietary habits were assessed using five questions from the Behavioral Risk Factor Survey (BRFS). Participants reported their average daily intake of (1) fruit and vegetables (none, 1–2, 3–4, or ≥5 servings), (2) fruit/vegetable juice (none, 1–2, 3–4, or ≥5 cups), (3) red meat (none, 1–3, 4–6, or ≥7 servings), (4) white meat (none, 1–3, 4–6, or ≥7 servings), and (5) processed meat (none, 1–2, 3–4, or ≥5 days per week). All five variables were treated as ordinal categorical variables.

Physical pain: Physical pain was evaluated using two sets of indicators. First, participants were asked whether they had experienced any issues (aching, pain, discomfort) in the past three months in seven body regions: neck, shoulder, forearm/wrist, low back, hip, thigh/knee, and ankle/foot. This item was assessed with a single yes/no question. Second, for each region where pain was present, participants reported the duration of the problem (less than 1 day, 1–7 days, 8–30 days, more than 30 days, or every day).

Covariates included age, gender (male/female), monthly income (HK$30,000–HK$39,999; HK$40,000–HK$59,999; ≥HK$60,000; or “prefer not to indicate”), school type (secondary school vs. special school), and mental health status. Mental health was measured using the Chinese version of the Depression Anxiety Stress Scales-21 (DASS-21) and the Pittsburgh Sleep Quality Index (PSQI). The DASS-21 comprises three subscales (depression, anxiety, stress), each with seven items scored on a four-point Likert scale (0 = not at all to 3 = most of the time). The PSQI assesses sleep quality over the preceding month, with a global score of >5 indicating poor sleep.

Statistical analyses: Demographic characteristics and all survey data were aggregated and summarized as descriptive statistics. Means and standard deviations (SDs) were presented in their corresponding contexts. Medians and interquartile ranges were reported in tables for continuous variables. Absolute numbers and relative frequencies from the total number of participants were presented for categorical variables. Independent t-test or one-way analysis of variance (ANOVA) was conducted to assess whether OSI-2 differed across the levels of nutritional health and the presence of physical pain (both categorical variables). Significant findings in ANOVA were further verified through linear regression to account for covariates, and the regression models were adjusted for multiple comparisons using Bonferroni correction. The covariates included in the models were age, gender, income, school type (mainstream secondary school versus special education school), mental health status, and sleep quality. We also performed cross-sectional path analysis to examine the potential pathway associations between depression, diet/pain, and stress using the PROCESS Macro Model 4 Version with 5000 bootstrap samples. As these data are cross-sectional and do not establish temporal precedence, this analysis assesses concurrent indirect pathways rather than causal mediation mechanisms. All tests were two-tailed, and a *p*-value < 0.05 was considered statistically significant. All data analyses were performed with IBM SPSS Statistics version 29.0 (SPSS Inc., Chicago, IL, USA).

## 3. Results

### 3.1. Participant Characteristics

Between June 2022 and June 2024, 671 teachers were invited to participate in the MED project, and 267 (65.5% female; mean [SD] age, 41.47 [10.99] years) completed our survey without missing data for the variables of interest and were thus included in the analysis. Most teachers (237, 88.8%) came from secondary schools. Notably, 30 teachers (11.2%) were from special schools. The mean OSI-2 score was 129.42 (SD = 44.50) with no significant difference between school types (*p* = 0.400). Full sociodemographic details are presented in Table 1.

### 3.2. Nutritional Health

**Figure 1:**
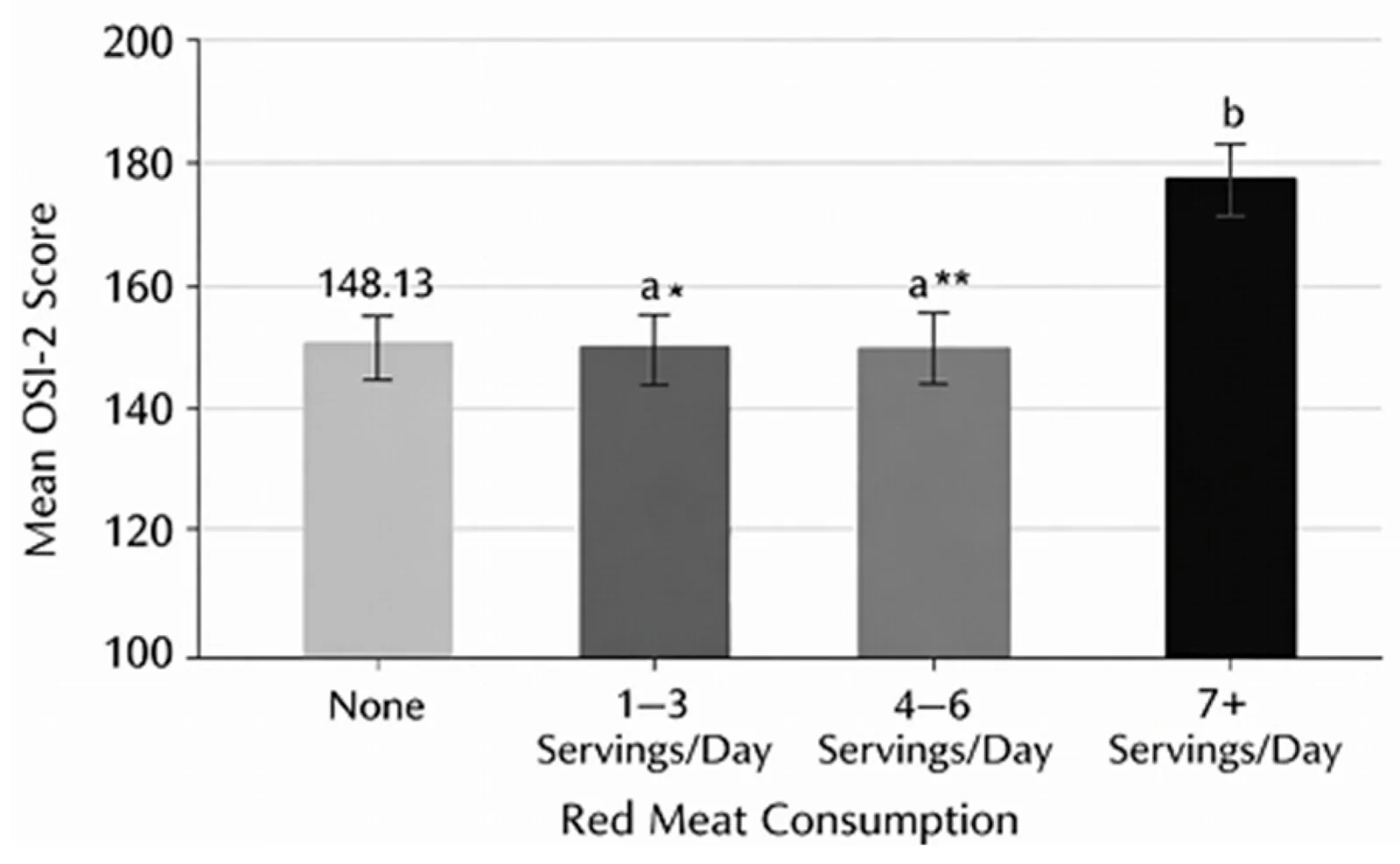
Mean OSI-2 scores by red meat consumption. Note: Error bars represent standard errors. * *p* < 0.05, ** *p* < 0.01. Different lowercase letters indicated statistically significant differences.

We first examined whether occupational stress (OSI-2) differed across levels of five dietary variables. A one-way ANOVA revealed a statistically significant difference in OSI-2 across the four levels of daily red meat consumption (F[3, 263] = 4.422, *p* = 0.005, 95% CI [0.005, 0.098]). The assumptions for ANOVA were examined. The Shapiro–Wilk test indicated that OSI-2 scores were normally distributed across three of the four levels of daily red meat consumption (*p* > 0.05), apart from teachers who consumed one to three servings of red meat per day (*p* = 0.001). However, given the robustness of ANOVA to moderate violations of normality, the analysis was continued. Levene’s test of equality of variances was not significant, F (3, 263) = 4.422, *p* = 0.947, supporting the assumption of homogeneity of variances across the four levels of daily red meat consumption. Thus, the assumptions for ANOVA were considered adequately met. Post hoc comparisons using the Bonferroni correction showed that teachers who consumed one to three servings of red meat per day (mean difference = −43.70, 95% CI [−83.33, −4.06], *p* = 0.022) and those who consumed four to six servings per day (mean difference = −50.15, 95% CI [−91.59, −8.70], *p* = 0.009) had significantly lower OSI-2 scores than teachers who consumed seven or more servings per day. Teachers who reported no red meat consumption (*n* = 15) had a mean OSI-2 score of 148.13 (SD = 43.38), which was not significantly different from any other group in the post hoc tests. The mean OSI-2 scores for the four groups are illustrated in Figure 1.

By contrast, no significant differences in OSI-2 were observed for the highest versus the lowest fruit and vegetable intake (≥5 servings versus none) (F[3, 263] = 0.422, *p* = 0.737, η^2^ = 0.005), fruit/vegetable juice consumption (≥5 cups versus none) (F[3, 263] = 0.403, *p* = 0.751), white meat consumption (≥7 servings versus none) (F[3, 263] = 2.111, *p* = 0.099), or processed meat frequency (≥5 days per week versus none) (F[3, 263] = 1.355, *p* = 0.257). These nonsignificant findings suggest that the association between red meat and occupational stress is specific rather than reflecting a general pattern of unhealthy eating. Complete results for these variables are provided in Table 2.

We accounted for potential confounders by conducting a multiple linear regression analysis with OSI-2 as the dependent variable and red meat consumption as the predictor. The analysis adjusted for age, gender, income, school type, DASS-depression, DASS-anxiety, DASS-stress, and PSQI. The model was statistically significant (F[9, 257] = 7.7, *p* < 0.001, R^2^ = 0.212, Adjusted R^2^ = 0.185), thus indicating that the set of predictors explained approximately 21.2% of the variance in OSI-2. Red meat consumption was not a significant predictor (B = 0.022, *p* = 0.696), while among the covariates, high stress scores (B = 0.311, 95% CI [6.256, 19.702], *p* < 0.001) were associated with elevated OSI-2. Meanwhile, depression, anxiety, sleep quality, and demographic variables were not significant (Table 3). Collinearity diagnostics indicated no multicollinearity concerns (all VIF < 5). These findings suggest that high stress scores are associated with higher OSI-2 scores.

### 3.3. Physical Pain

#### 3.3.1. Physical Pain-Specific Analysis

We then examined OSI-2 differences according to the presence of pain in seven body regions. Teachers with neck pain (*n* = 191, M = 132.69, SD = 44.47) had higher OSI-2 scores than those without (*n* = 76, M = 121.20, SD = 43.79). This difference was close to significance for the two-sided test (*p* = 0.057). Moreover, the effect size was small (Cohen’s d = 0.26, 95% CI [−0.01, 0.53]). Meanwhile, significant differences were found for three regions (Table 4). Teachers with shoulder pain (*n* = 180, M = 133.32, SD = 44.66) reported higher OSI-2 scores than those without (*n* = 87, M = 121.33, SD = 43.31; t[265] = 2.076, *p* = 0.039, Cohen’s d = 0.271). Similarly, teachers with forearm/wrist pain (*n* = 75, M = 141.29, SD = 41.00) scored higher than those without (*n* = 192, M = 124.78, SD = 45.05; t[265] = 2.76, *p* = 0.006, Cohen’s d= 0.38). Teachers with hip pain (*n* = 53, M = 140.68, SD = 43.66) also scored higher than those without (*n* = 214, M = 126.63, SD = 44.36; t[265] = 2.07, *p* = 0.039, Cohen’s d = 0.32). No significant differences in OSI-2 were observed for low back pain, thigh/knee pain, or ankle/foot pain (all *p* > 0.05) (Table 5).

#### 3.3.2. Pain Duration Analysis

For each body region where pain was present, we examined whether the duration of pain (less than 1 day, 1–7 days, 8–30 days, more than 30 days, or every day) was associated with OSI-2 using one-way ANOVA. For all seven regions, the overall ANOVA results were nonsignificant (all *p* > 0.059, Table 6). For shoulder and hip pain, the “more than 30 days” group had numerically higher OSI-2 scores (e.g., shoulder: M = 152.71, SD = 33.02; hip: M = 158.63, SD = 36.59). However, these differences were not statistically significant (Supplementary Table S1).

#### 3.3.3. Multivariable Linear Regression and Path Analysis

We accounted for potential confounders to conduct a multiple linear regression analysis with OSI-2 as the dependent variable and neck pain duration as the predictor. The analysis adjusted for age, gender, income, school type, DASS-depression, DASS-anxiety, DASS-stress, and PSQI. The model was statistically significant (F[9, 181] = 7.681, *p* < 0.001, R^2^ = 0.276, Adjusted R^2^ = 0.240), thus indicating that the set of predictors explained approximately 27.6% of the variance in OSI-2. Neck pain duration was not a significant predictor (B = −0.084, *p* = 0.222), while among the covariates, high stress scores (B = 0.325, 95% CI [6.093, 22.358], *p* < 0.001) were associated with elevated OSI-2. Meanwhile, depression, anxiety, sleep quality, and demographic variables were not significant. Collinearity diagnostics indicated no multicollinearity concerns (all VIF < 10). These findings suggest that high stress scores are associated with higher OSI-2 scores (Table 7).

The regression results indicated that neck pain duration was not significantly related to depression (B = 0.057, SE = 0.035, t = 1.602, *p* = 0.111, 95% CI [−0.013, 0.126]). Controlling for neck pain duration and covariates, depression was not a statistically significant predictor of occupational stress at the α = 0.05 level, though it showed a marginal positive trend (B = 8.472, SE = 4.659, t = 1.818, *p* = 0.071, 95% CI [−0.722, 17.665]). The direct path linking neck pain duration to occupational stress was nonsignificant (B = −2.735, SE = 2.233, t = −1.225, *p* = 0.222, 95% CI [−7.141, 1.672]), as was the total association (B = −2.256, SE = 2.232, t = −1.011, *p* = 0.313, 95% CI [−6.659, 2.147]). The concurrent indirect path from neck pain duration to occupational stress through depression was evaluated using 5000 bias-corrected bootstrap samples and was not statistically significant (B = 0.479, BootSE = 0.435, 95% Boot CI [−0.047, 1.809]).

## 4. Discussion

In this cross-sectional study of 267 Hong Kong schoolteachers, we found that elevated occupational stress was significantly associated with prolonged musculoskeletal pain (duration > 30 days) even after adjusting for mental health and demographic covariates. These findings extend our understanding of the physical and nutritional health correlates of teacher stress beyond the previously documented mental health effects.

### 4.1. Red Meat Consumption and Occupational Stress

In our analysis, none of the dietary variables, including red meat, white meat, processed meat, fruits, or vegetables, were significantly associated with occupational stress as measured by the OSI-2. While the literature has previously reported associations between stress and various dietary factors, such as increased cravings for high-fat, energy-dense foods [19,20,21], we did not observe these relationships in our sample. The lack of significant findings may be attributable to several factors. First, our limited sample size may have reduced statistical power to detect modest but meaningful effects. Second, for our teacher population, physical pain or other occupational health issues may be more salient and immediate concerns than dietary patterns when it comes to stress manifestation. These null findings highlight that the determinants of occupational stress and their interaction with dietary behaviors may be population-specific, and that findings from one cultural or occupational context do not necessarily generalize to others. Further research with larger, more diverse samples is needed to clarify these relationships.

### 4.2. Musculoskeletal Pain and Occupational Stress

Our univariable findings that shoulder, forearm/wrist, and hip pain are associated with high OSI-2 are consistent with a large body of evidence linking work-related stress to musculoskeletal disorders [13,15]. The prevalence of pain in our sample (shoulder 67.4%, forearm 28.1%, hip 19.8%) is comparable with that reported among teachers in mainland China [14]. The effect sizes (Cohen’s d = 0.32–0.54) are within the range reported in the meta-analyses of psychosocial work stressors and musculoskeletal outcomes. However, after adjusting for covariates, particularly depression, anxiety, and stress symptoms, none of the specific pain locations remained significant. Only pain duration exceeding 30 days was independently associated with higher OSI-2. When interpreting the pain analysis results, particularly for shoulder and hip pain, several factors must be considered. Although numerical differences in OSI-2 scores were observed between duration groups, these did not reach statistical significance. One potential explanation is limited statistical power resulting from the small sample sizes in these specific pain categories (*n* = 8–17), which increases the risk of a Type II error and may mask true underlying differences. Alternatively, these nonsignificant results may indicate that duration of pain does not substantially impact OSI-2 scores in this population, and that the null hypothesis holds true. Future studies utilizing larger, adequately powered samples are required to clarify whether these observed numerical trends reflect true clinically meaningful differences or random variation. This pattern suggests that the relationship between site-specific pain and occupational stress is largely confounded or mediated by mental health, particularly depression, which was the strongest predictor in our linear regression model (β = 0.079, *p* = 0.002). The bidirectional link between depression and chronic pain is well established [18]. Mechanistically, depression and chronic pain share overlapping neurobiological pathways, including dysregulation of neurotransmitter networks (e.g., glutamate, gamma-aminobutyric acid, and monoamines), activation of inflammatory cytokines such as tumor necrosis factor-alpha and interleukin-6, and structural and functional changes in brain regions involved in emotion regulation and pain processing, including the prefrontal cortex and anterior cingulate cortex [22,23]. Psychologically, depression may lower pain thresholds, increase pain catastrophizing, and reduce self-efficacy for pain management, while chronic pain itself contributes to depressive symptoms through functional limitations, sleep disruption, and social withdrawal [22].

### 4.3. Comparison with the Previous Literature

To our knowledge, this work is the first study to examine red meat consumption and multiple pain sites simultaneously in relation to occupational stress among Hong Kong teachers. Our findings add to the small number of studies on teacher stress in non-Western settings. A systematic review of musculoskeletal disorders among teachers confirmed that factors such as awkward posture, prolonged standing, and sitting are associated with higher MSD prevalence [13]. Our results suggest that stress may operate partly through these biomechanical pathways.

### 4.4. Limitations

Several limitations should be acknowledged. First, the cross-sectional design prevents causal inference. Second, dietary and pain data were self-reported and subject to recall bias. Third, the sample was predominantly from secondary schools (89%), thus limiting generalizability to special schoolteachers or primary school teachers. Fourth, our reliance on self-reported dietary intake, pain presence/duration, and occupational stress may introduce recall and social desirability biases. We did not collect objective biomarkers (e.g., salivary cortisol, serum C-reactive protein, or other inflammatory markers) or obtain clinical diagnoses of musculoskeletal conditions. The inclusion of such objective measures in future research would strengthen causal inference and validate our self-report findings. Fifth, we employed complete-case analysis, which may introduce selection bias if missingness is not completely random. During the COVID-19 pandemic, a substantial proportion of participants had missing data on key variables, and imputation would have made the results highly dependent on model assumptions. Sixth, our pain duration analyses were limited by small subgroup sample sizes (as few as *n* = 8 in the >30 days category for some regions), which may have led to unstable estimates and reduced statistical power. Lastly, additional potential confounders, such as physical activity levels, body mass index, and social support, were not collected.

### 4.5. Implications for Practice and Future Research

Our results have several practical implications. Occupational health programs for teachers should address stress-related eating behaviors by offering nutritional counseling and promoting a workplace-friendly healthful diet (e.g., plant-based proteins, fish). As a result, access to nutritional experts should be made available in schools. On the other hand, given that prolonged pain (>30 days) was associated with high stress even after mental health adjustment, schools should provide ergonomic assessments and access to physiotherapy for teachers with persistent musculoskeletal symptoms. The strong role of depression suggests that mental health support, such as cognitive behavioral therapy or mindfulness-based interventions, has demonstrated positive effects in reducing teacher stress. Such interventions delivered by occupational therapists and social workers or their trained assistants may be the most effective strategy for reducing overall stress. Notably, these strategies have potential spillover benefits for diet and pain. Longitudinal cohort studies with repeated measures are necessary to clarify the directionality of these relationships. Subgroup analyses by school type, gender, and teaching experience should be considered in large samples.

## 5. Conclusions

Occupational stress among Hong Kong schoolteachers is independently associated with prolonged musculoskeletal pain, with mental health, particularly depression, strongly confounding the pain–stress association. Since occupational stress not only affects mental health but might also link to lifestyle factors, schools should implement targeted interventions for teachers: (1) nutritional counseling with dietitians, (2) ergonomic assessments and physiotherapy for persistent pain, and (3) cognitive behavioral therapy with depression screening. This integrated approach addresses the direct physical, nutritional, and mental health consequences of occupational stress.

## Figures and Tables

**Table 1:** Sociodemographic characteristics.

Characteristic	Teachers in S1 (*n* = 237)	Teachers in S2 (*n* = 30)	Overall (*n* = 267)	*p*-Value
**Age** (**mean [SD]**)	41.41 [11.11]	41.97 [10.20]	41.47 [10.99]	0.794
**Gender**				
Male	83 (35.0%)	9 (30.0%)	92 (34.5%)	0.587
Female	154 (65.0%)	21 (70.0%)	175 (65.5%)	
**Income**				
HK$30,000–HK$39,999	44 (18.6%)	3 (10.0%)	47 (17.6%)	0.009
HK$40,000–HK$59,999	49 (20.7%)	7 (23.3%)	56 (21.0%)	
≥HK$60,000	131 (55.3%)	14 (46.7%)	145 (54.3%)	
Prefer not to indicate	13 (5.5%)	6 (20.0%)	19 (7.1%)	
**OSI-2**	128.6 [43.10]	135.87 [54.76]	129.42 [44.50]	0.400

Abbreviation: S1 = Secondary schools; S2 = Special schools.

**Table 2:** Occupational stress (OSI-2) differed across levels of four dietary variables.

Variable	F[df]	*p*	η^2^
**Fruit and vegetable intake**	F[3, 263] = 0.422	0.737	0.005
**Fruit/vegetable juice consumption**	F[3, 263] = 0.403	0.751	0.005
**White meat consumption**	F[3, 263] = 2.111	0.099	0.024
**Processed meat frequency**	F[3, 263] = 1.355	0.257	0.015

**Table 3:** Predictors of OSI-2 scores using red meat consumption as the predictor.

Predictor	B [95% CI]	*p*-Value
**DASS_stress**	0.311 [6.256, 19.702]	*p* < 0.001

**Table 4:** Significant differences in OSI-2 scores with or without physical pain.

Pain Region	Pain Status	*n*	M	SD	T[265)	*p*	Cohen’s d
Shoulder	With pain	180	133.32	44.66	2.076	0.039	0.271
	Without pain	87	121.33	43.31			
Forearm/wrist	With pain	75	141.29	41.00	2.760	0.006	0.38
	Without pain	192	124.78	45.05			
Hip	With pain	53	140.68	43.66	2.070	0.039	0.32
	Without pain	214	126.63	44.36			

**Table 5:** No significant differences in OSI-2 scores with or without physical pain.

Pain Region	Pain Status	*n*	M	SD	T[265)	*p*	Cohen’s d
Low back	With pain	166	130.04	42.18	0.292	0.771	0.037
	Without pain	101	128.40	48.27			
Thigh/knee	With pain	105	133.60	44.20	1.238	0.217	0.155
	Without pain	162	126.70	44.61			
Ankle/foot	With pain	87	136.37	40.63	1.782	0.076	0.233
	Without pain	180	126.06	45.98			

**Table 6:** Levels of OSI-2 scores by pain region.

Variable	F[df]	*p*	η^2^
Neck	F[4, 186] = 2.320	0.059	0.048
Shoulder	F[4, 175] = 0.980	0.420	0.022
Forearm/wrist	F[4, 70] = 0.320	0.863	0.018
Low back	F[4, 161] = 0.424	0.791	0.010
Hip	F[4, 49] = 1.214	0.317	0.090
Thigh/knee	F[4, 100] = 1.127	0.348	0.043
Ankle/foot	F[4, 82] = 2.088	0.090	0.092

**Table 7:** Predictors of OSI-2 scores using neck pain duration as the predictor.

Predictor	B [95% CI]	*p*-Value
**DASS_stress**	0.325 [6.093, 22.358]	*p* < 0.001

## Data Availability

The original contributions presented in this study are included in the article. Further inquiries can be directed to the corresponding author.

## Supplementary Materials

The following supporting information can be downloaded at: https://www.mdpi.com/article/10.3390/nu18182956/s1, Table S1: Differences in OSI-2 scores within or without physical pain.

## Abbreviations

The following abbreviations are used in this manuscript:

MSDs	Musculoskeletal Disorders
MED	Mental, Exercise, and Diet Health
BRFS	Behavioral Risk Factor Survey
OSI-2	Occupational Stress Indicator-2
DASS-21	Depression Anxiety Stress Scales-21
PSQI	Pittsburgh Sleep Quality Index
