# Supplementary material for: Occupational Stress, Nutritional Health, and Physical Pain Among Hong Kong Schoolteachers: A Cross-Sectional Study
Source: Nutrients. 2026 Sep 9;18(18):2956. doi: 10.3390/nu18182956 (PMC13610049; doi:10.3390/nu18182956)
Supplement: Supplementary file 1 [file nutrients-18-02956-s001.zip › nutrients-4429898-supplementary.pdf]

Supplementary Table S1. Differences in OSI-2 scores within or without physical pain.

Pain Region	Pain duration	n	M	SD
Neck	Less than 1 day	15	127.53	45.346
	1–7 days	96	126.70	46.061
	8–30 days	37	142.51	38.720
	More than 30 days	22	153.14	36.040
	Every day	21	125.00	48.100
Shoulder	Less than 1 day	13	130.31	48.902
	1–7 days	88	129.51	47.873
	8–30 days	38	134.34	36.560
	More than 30 days	17	152.71	33.015
	Every day	24	133.58	48.529
Forearm/wrist	Less than 1 day	8	153.88	35.490
	1-7 days	39	138.10	43.088
	8-30 days	11	146.00	30.037
	More than 30 days	6	145.17	46.735
	Every day	11	136.64	47.806
Low back	Less than 1 day	15	132.60	39.417
	1–7 days	75	128.07	42.996
	8–30 days	36	131.03	37.844
	More than 30 days	25	138.00	39.802
	Every day	15	121.67	55.903
Hip	Less than 1 day	3	135.33	38.136
	1–7 days	20	149.05	38.899
	8–30 days	15	132.60	31.899
	More than 30 days	8	158.63	36.594
	Every day	8	118.63	70.614
Thigh/knee	Less than 1 day	7	150.00	52.083
	1–7 days	41	127.17	43.269
	8–30 days	30	132.13	35.907
	More than 30 days	10	156.10	32.532
	Every day	17	131.71	59.348
Ankle/foot	Less than 1 day	6	174.50	22.421
	1–7 days	38	134.87	39.652
	8–30 days	24	124.25	35.942
	More than 30 days	9	141.00	53.463
	Every day	10	144.10	41.605
